# Quality Analysis of YouTube-Based Exercise Programs for Typically Developing Children: Content Analysis

**DOI:** 10.3390/healthcare13050560

**Published:** 2025-03-05

**Authors:** Juntaek Hong, Yerim Do, Dong-wook Rha, Na Young Kim

**Affiliations:** 1 Department and Research Institute of Rehabilitation Medicine, Yonsei University College of Medicine, Seoul 03722, Republic of Korea; ghdwnsxor@yuhs.ac (J.H.); doyr62@yuhs.ac (Y.D.); medicus@yonsei.ac.kr (D.-w.R.); 2 Department of Rehabilitation Medicine, Yongin Severance Hospital, Yonsei University College of Medicine, Yongin 16995, Republic of Korea; 3 Center for Digital Health, Yongin Severance Hospital, Yonsei University College of Medicine, Yongin 16995, Republic of Korea

**Keywords:** YouTube, child, exercise program, usability, video quality

## Abstract

**Background:** Physical activities in childhood are important. However, a lack of exercise among children and adolescents is becoming a global reality. Moreover, following the coronavirus disease 2019 pandemic, the increase in time spent at home has led to qualitative changes, such as at-home exercises and the use of YouTube content. This study aimed to conduct qualitative assessments of YouTube-based exercise education programs, such as video content and exercise education programs. **Methods:** A Python-based (version 3.11.6) video data crawl of YouTube using the keywords “children + exercise”, “kid + exercise”, “child + physical activity”, and “kid + physical activity” was conducted on 27 November 2023. Duplicate, non-English, outdated (over 5 years old), short (<60 s) or long (>30 min) videos, and irrelevant content were excluded. Basic video characteristics, video popularity metrics, and qualitative analyses (m-DISCERN, GQS, i-CONTENT, CONTENT, CERT) were collected and assessed. **Results:** Of the 2936 retrieved videos, 126 were selected. Approximately 10% of the videos were uploaded by health professionals, and most videos covered aerobic and muscle-strengthening exercises. A qualitative analysis of the video content showed moderate to high quality, while only a few videos satisfied the criteria of an effective exercise program, especially in terms of “Type and timing of outcome assessment”, “Qualified supervisor”, “Patient eligibility”, “Adherence to the exercise program”, and “Dosage parameters (frequency, intensity, time)”. In the correlation analysis of video content and exercise program quality, only a few items showed a statistically significant correlation. **Conclusions:** YouTube exercise-related educational content targeting children may be inadequate and is not correlated with video popularity. Although an overall weak to moderate correlation was observed between the quality evaluation of exercise education and video content, the use of video quality assessment tools to evaluate exercise program quality was insufficient.

## 1. Introduction

The importance of physical activity during childhood cannot be overemphasized. Maintaining optimal exercise levels during childhood and adolescence reduces cardiometabolic risk and promotes bone density and growth [1]. Moreover, it is known to have positive effects not only on physical health but also on cognitive function and mental health [2]. In the long term, physical activity during childhood is closely related to that during adolescence and adulthood [3].

Given the importance of physical activity behavior, the American College of Sports Medicine guidelines strongly recommend performing exercise for more than 1 h, three to five times per week [4]. However, the lack of exercise among children and adolescents is becoming a global issue. According to the previous study based on self-report data, about 80% of 11–17-year-olds were physically inactive [5]. Additionally, accelerometry data from American and European youth aged 10–24 revealed a sharper decline in physical activity as adolescents aged [6]. Along with this trend, the mean BMI and obesity rates in children and adolescents aged 5–19 years have increased in most regions and countries, highlighting the urgent need for well-structured strategies to promote physical activity behaviors in children and adolescents [7].

Meanwhile, following the onset of the coronavirus disease 2019 (COVID-19) pandemic, discernible shifts were observed in the qualitative and quantitative dimensions of physical activity trends for children, reflecting an increase in screen time at home and a decrease in spontaneous outdoor activities [8]. Correspondingly, YouTube-based fitness content has garnered significant attention. However, there are some concerns about their clinical effectiveness [9].

There have been several prior studies of the effects of web-based exercise programs, including YouTube content; however, their clinical efficacy remains controversial [10,11]. Unlike offline educational programs, no previous study has evaluated the qualitative aspects of web-based exercise programs. With digital tools becoming common in education, considering their potential impact on children’s health, evaluating the quality of such information is essential. Consequently, this study aimed to perform a comprehensive qualitative and quantitative analysis of YouTube-based educational exercise content for children, including an analysis of the association between video content quality and educational program quality.

## 2. Methods

### 2.1. Study Design and Video Selection Strategy

**Figure 1:**
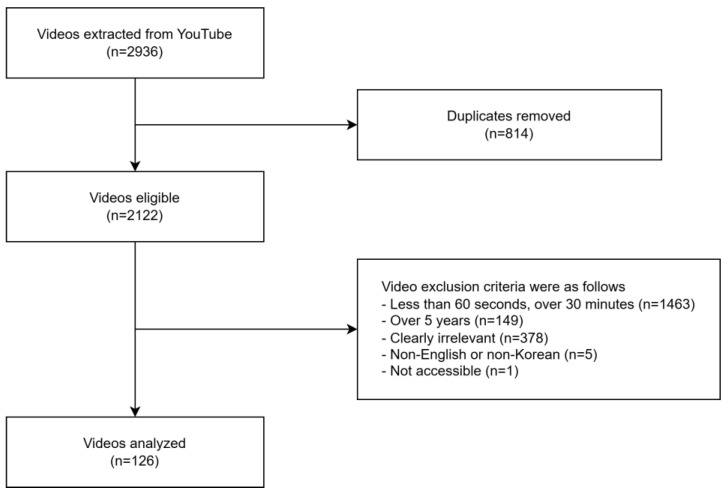
Flowchart demonstrating video selection process.

YouTube was scoured for relevant content on 27 November 2023 using general terms that were easily searched, even for individuals with limited knowledge of types of exercises, as follows: “children + exercise”, “kid + exercise”, “child + physical activity”, and “kid + physical activity”. Videos were selected using a Python-based crawling technique (version 3.11.6) with specific keywords and criteria to ensure the inclusion of all available videos. The exclusion criteria were as follows: duplicate and non-English-language videos uploaded more than 5 years before the search date, based on the criteria of previous studies [12]; play time < 60 s to exclude short-form videos inappropriate for information delivery purposes [13] or >30 min, which were used as exclusion criteria in previous studies targeting in-depth information [14,15]; and inappropriate content on exercise and physical activities from an educational perspective, such as videos lacking audio for the purpose of imparting knowledge, consisting solely of raw footage or photographs without explanations, or created for advertising purposes. Videos deemed inappropriate by the researchers were also omitted. Figure 1 shows the video selection process, while Table S1 lists the specific reasons for excluding each video. Our study exclusively utilized publicly available online videos, with no direct interaction involving humans or animals, and no collection or use of personally identifiable information. Hence, approval from an ethical committee was not necessary.

### 2.2. Video Assessment

The basic characteristics of each video, including title, uniform resource locator, uploader, upload date, and exercise type, were collected. The classification of exercise types adhered to the recommendations outlined in the Physical Activity Guidelines for Americans [4], distinguishing between aerobic and strength-training exercises. Videos featuring both types of exercises were categorized as “multicomponent”, whereas those not aligned with either were classified as “other”. Furthermore, two researchers (JH and YD), each with over 5 years of extensive experience in physical exercise and pediatric rehabilitation, conducted a comprehensive assessment of the videos across three domains: video popularity-related parameters, qualitative assessment tools of video content, and exercise programs. The researchers resolved discrepancies through discussion and consensus.

### 2.3. Quantitative Information and Video Popularity-Related Parameters

Quantitative data pertaining to the video content, including playtime, number of likes, dislikes, and total views, were gathered. Using this information, the Video Power Index (VPI) was computed as a metric of video popularity. This index is derived by dividing the product of the like-to-view ratio by 100. The like-to-view ratios were calculated as follows: (number of likes × 100)/(total number of likes and dislikes) and (number of total views/days since upload) [12].

### 2.4. Quality Assessment Tools

This study conducted a qualitative analysis from two perspectives: video content quality and exercise program quality. All the assessment tools used in this study were either developed through expert consensus in the relevant field [16], including the Delphi method [17,18], or selected from tools that have been widely used in video content quality analysis across numerous previous studies as below.

#### 2.4.1. Video Content Quality

A quality assessment of the video content was conducted using the modified DISCERN (m-DISCERN) tool and the global quality scale (GQS) [19,20]. The m-DISCERN, a widely used evaluation index for assessing video content reliability, comprises five items rated on a binary scale. A higher score indicates greater reliability, with a threshold of ≥3 out of a maximum score of 5 indicating significant reliability [21]. Furthermore, the overall video quality was appraised using the 5-point GQS, which was designed to assess the quality and flow of online information [22]. Overall ratings of 4–5 points were designated as high, 3 points as moderate, and 1–2 points as low quality [23]. Moreover, to clarify the scores obtained for each quality and flow section, each section was divided into poor, moderate, and good groups based on the GQS score criteria.

#### 2.4.2. Exercise Program Quality

The quality assessment of the exercise programs used the International CONsensus on Therapeutic Exercise aNd Training (i-CONTENT) tool [24], CONsensus on Therapeutic Exercise aNd Training (CONTENT) scale, and Consensus on Exercise Reporting Template (CERT) [25]. The i-CONTENT tool comprises seven items assessed on a binary scale. The scores obtained from this tool facilitate classification into low, moderate, and high risk of ineffectiveness categories [26]. The CONTENT scale comprises a total of nine items categorized into five sections aimed at evaluating the therapeutic validity of the exercise. Each section was assessed using a binary scoring system; a score of ≥6 out of 9 indicates high therapeutic quality [27]. In contrast, the CERT is a semiquantitative scoring tool that assesses the completeness of exercise descriptions using 16 binary items, with higher scores indicating more comprehensive content [28].

### 2.5. Statistical Analysis

Descriptive data are shown as numbers, percentages, medians, and interquartile ranges. The Shapiro–Wilk test was used to assess the normality of the data distribution. Cochran’s Q and McNemar’s tests were performed to compare the differences between the proportions of items, and a Bonferroni test was used for multiple-comparison correction. Pearson’s correlation analysis was conducted to examine the association among the assessments. Cohen’s weighted kappa or Spearman’s rank correlation analysis was used to assess the strength and direction of the relationship between the binary nominal or ordinal categorical data. The scales of the two coefficients were based on the ranges presented previously [29,30]. Statistical significance was set at *p* < 0.05, and all analyses were performed using the RStudio software (R version 4.1.3).

## 3. Results

### 3.1. General Characteristics and Quantitative Analysis of Video Content

Out of the 2936 videos via YouTube search, 126 met the inclusion criteria after applying exclusions (Figure 1). The basic characteristics of the videos are listed in Table 1. Only 10.31% of the videos were uploaded by health professionals who confirmed their affiliation with and possession of a health-related certificate. The largest proportion of exercise type was multicomponent exercise (84.13%). A detailed classification of exercise types is provided in Table S2.

### 3.2. Qualitative Assessment of Video Content

**Figure 2:**
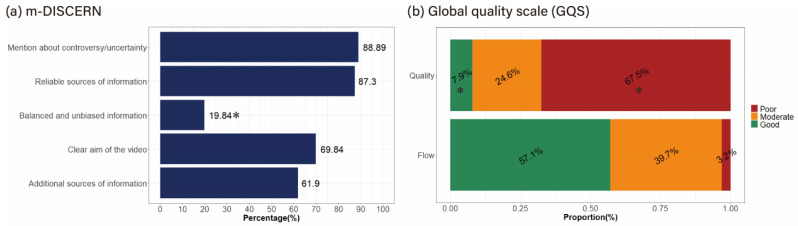
Satisfaction analysis between items of quality assessment tools of video content. Data were analyzed using McNemar test, and Bonferroni test was used for multiple-comparison correction. * *p* < 0.001. Abbreviation: m-DISCERN, modified DISCERN; GQS, global quality scale.

Table 2 summarizes the results of the quality analysis of the video content. For the classification of m-DISCERN based on the obtained scores, 81.75% were classified as “reliable”. For m-DISCERN, the uncertainty (“Are areas of uncertainty mentioned?”) and reliability (“Are reliable sources of information used?”) items were satisfied by >80% of the video contents, while the items about the aim of the video contents (“Are the aims clear and achieved?”) and presence of additional information (“Are additional sources of information listed for patient reference?”) were satisfied by 60–70% of all video contents. However, the balance item (“Is the information presented both balanced and unbiased?”) was satisfied by <20%, a value that was significantly lower than the other items (*p* < 0.001) (Figure 2a).

The mean GQS score was 3.29 ± 1.01 (maximum 5), indicating quality and flow between the moderate/suboptimal (GQS score of 3) and good (GQS score of 4). For the classification of GQS based on the total scores obtained, contents classified as high, moderate, and low quality accounted for 23.80%, 42.06%, and 34.12%, respectively. The analysis of video content flow and quality revealed a significantly higher proportion of “good quality” than “good flow” (57.1% vs. 7.9%, respectively; *p* < 0.001). In contrast, “poor flow” showed a statistically significantly higher proportion than “poor quality” (67.5% vs. 3.2%, respectively; *p* < 0.001) (Figure 2b).

### 3.3. Qualitative Assessment of Exercise Program

Table 3 summarizes the results of the quality analyses of the exercise programs. The mean i-CONTENT, CONTENT, and CERT scores were 2.62 ± 0.77 (of 7), 1.53 ± 1.25 (of 9), and 4.50 ± 2.36 (of 19), respectively. For the quality classification, according to i-CONTENT, not a single video fell into the “low risk of ineffectiveness” category, while the vast majority, 93.65%, belonged to the “high risk of ineffectiveness” category. Similarly, according to CONTENT, 98.41% of the videos were classified as “ineffective”.

**Figure 3:**
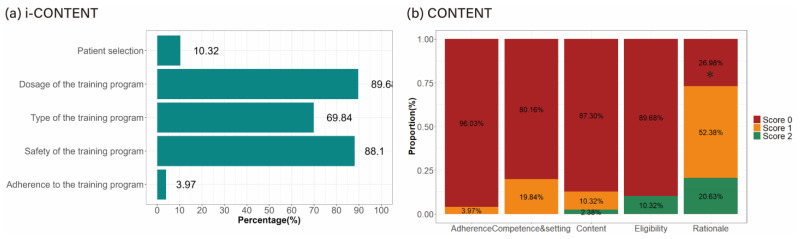
Satisfaction analysis among items of quality assessment tools of exercise program. Data were analyzed using McNemar test, and Bonferroni test was used for multiple-comparison correction. * *p* < 0.001. Abbreviation: i-CONTENT, International CONsensus on Therapeutic Exercise aNd Training; CONTENT, CONsensus on Therapeutic Exercise aNd Training.

In the i-CONTENT subcategory analysis, none of the videos’ contents were satisfactory in the categories of “Qualified supervisor” or “Type and timing of outcome assessment”. Moreover, the items “Patient selection” (10.32%; *p* < 0.001) and “Adherence to the exercise program” (3.97%; *p* < 0.001) showed statistically significantly lower proportions than the other three items (Figure 3a). The items “Dosage parameters (frequency, intensity, time)”, “Type of exercise”, and “Safety of the exercise program” were satisfied by approximately 89.68%, 69.84%, and 88.1%, respectively.

For CONTENT, “Rationale” (73.01%) was the only section that achieved 50% satisfaction with at least one item; this proportion was significantly higher than those of the other four sections (*p* < 0.001) of “Patient eligibility”, “Competences and setting”, “Content”, and “Adherence” at 10.32%, 19.84%, 12.70%, and 3.97%, respectively (Figure 3b).

### 3.4. Correlation Study of Analyses

#### 3.4.1. Quantitative Assessment of Video Content and Other Qualitative Assessments

Qualitative parameters related to the video content and exercise education programs did not show any statistical correlation with the quantitative parameters of the video content. In particular, no statistically significant association was observed with VPI, the parameter most closely related to popularity (Table 4).

#### 3.4.2. Comparison of Qualitative Assessments

**Figure 4:**
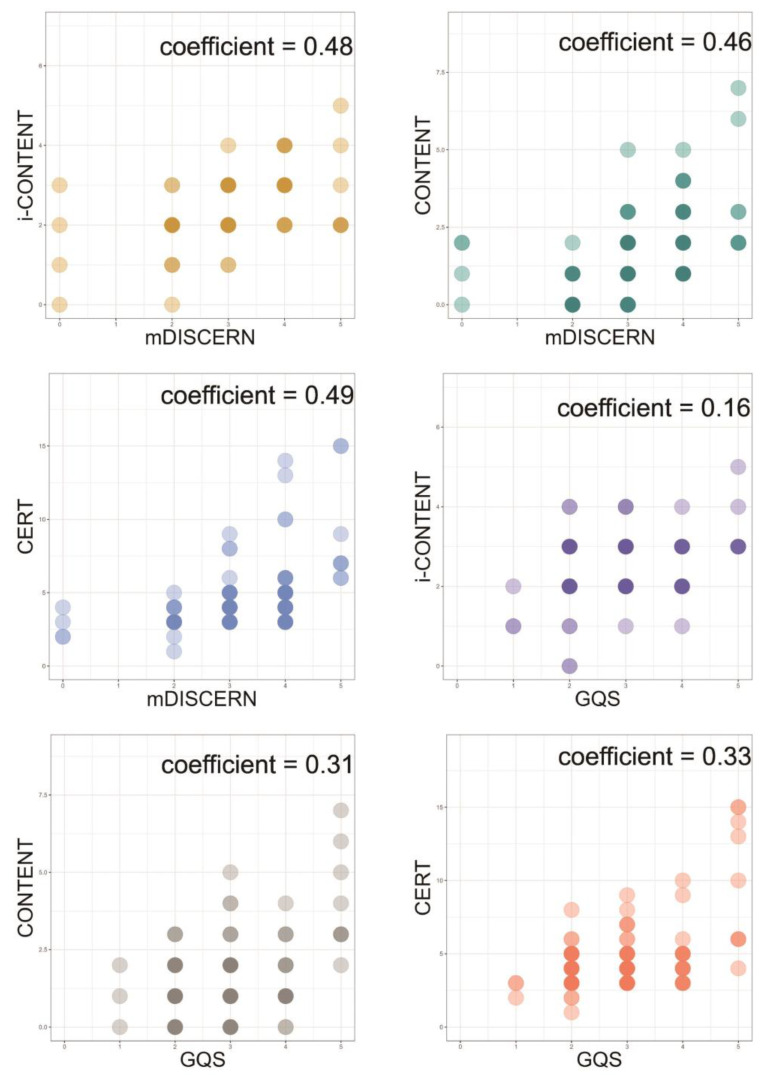
Correlation plot for qualitative assessment between exercise program and video content. Spearman rank correlation analysis was used in this figure. Abbreviation: m-DISCERN, modified DISCERN; GQS, global quality scale; i-CONTENT, International CONsensus on Therapeutic Exercise aNd Training; CONTENT, CONsensus on Therapeutic Exercise aNd Training; CERT, Consensus on Exercise Reporting Template.

Statistical analysis found a moderate correlation between m-DISCERN scores and all exercise education assessment tools (ρ: i-CONTENT, 0.48; CONTENT, 0.46; and CERT, 0.49), whereas GQS scores showed only a weak positive correlation with CONTENT (ρ = 0.31) and CERT (ρ = 0.31) and no significant link with i-CONTENT (ρ = 0.16) (Figure 4).

**Figure 5:**
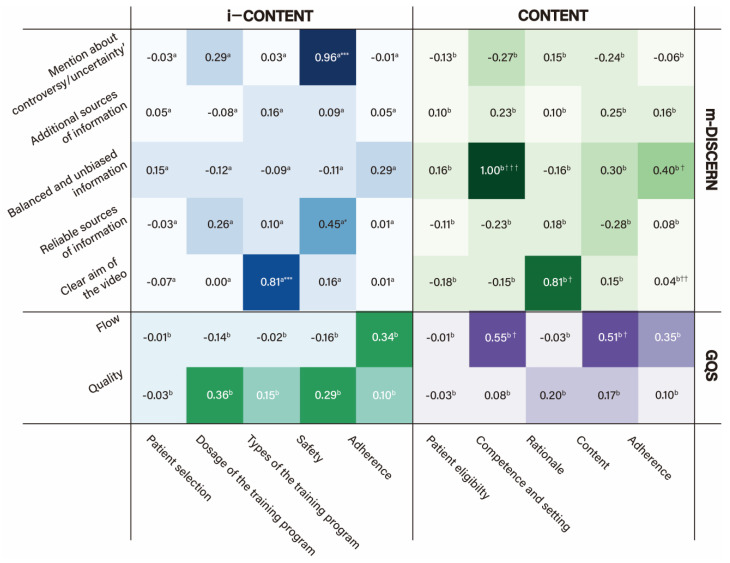
Heatmap for qualitative assessment between exercise program and video content. ^a^ Cohen’s kappa coefficient, ^b^ Spearman rank correlation coefficient, † moderate correlation in Spearman rank correlation analysis, †† high correlation in Spearman rank correlation analysis, ††† almost perfect correlation in Spearman rank correlation analysis, * moderate agreement in Cohen’s kappa coefficient, and *** almost perfect agreement in Cohen’s kappa coefficient. Abbreviation: m-DISCERN, modified DISCERN; GQS, global quality scale; i-CONTENT, International CONsensus on Therapeutic Exercise aNd Training; CONTENT, CONsensus on Therapeutic Exercise aNd Training.

Figure 5 shows the correlation between the qualitative assessment items of the exercise program and video content. In the correlation analysis between items of m-DISCERN for video quality and those of i-CONTENT and CONTENT for exercise program quality, only two from i-CONTENT and three from CONTENT showed statistically significant correlations with those of m-DISCERN. For items that showed statistical correlations, “Safety of the exercise program” for i-CONTENT showed almost perfect agreement with the uncertainty item (“Are areas of uncertainty mentioned?”) (κ = 0.96) and moderate agreement with the reliability item (“Are reliable sources of information used?”) (κ = 0.45) for m-DISCERN. Moreover, the balance item (“Is the information presented both balanced and unbiased?”) for m-DISCERN showed a very high correlation with “Competences and setting” for CONTENT (ρ = 1.00). “Clear aim of the video” for m-DISCERN also showed almost perfect agreement with “Type of the exercise program” for CONTENT (κ = 0.81) and a very high correlation with “Rationale” for CONTENT (ρ = 0.81). In the correlation analysis between items on the GQS for video quality and two exercise program quality evaluation tools, all items for i-CONTENT showed no significant correlation with GQS sections, while only two items for CONTENT, “Competences and setting” (ρ = 0.55) and “Content” (ρ = 0.51), showed moderate correlations with the flow section of the GQS.

## 4. Discussion

### 4.1. Principal Results

To the best of our knowledge, this research provides the first comprehensive qualitative and quantitative assessment of YouTube-based educational exercise content for children, including an analysis of the statistical relationships between video content quality and educational program quality. There was a lack of uploader information, which affected reliability, along with challenges in analyzing exercise types due to the combination of muscle strengthening and aerobic exercises in most of the included content. Previous studies have shown that expert-provided videos had higher reliability and quality [31,32], indicating the need for more professional health provider involvement in pediatric physical education content.

The qualitative analysis of video content revealed moderate quality and suboptimal flow. However, the balance item for m-DISCERN and the quality-related section of the GQS showed significantly lower satisfaction levels than the other items. This phenomenon may be influenced by the inherent nature of YouTube video content, in which important information must be delivered in a short time to gain popularity. Additional efforts, such as the use of YouTube scripts, could be needed to access relevant information.

In the qualitative analysis of the exercise education program, no videos met items for i-CONTENT about the outcome assessment and presence of a qualified supervisor, and adherence-related items for i-CONTENT and CONTENT scored less than 5%. These areas have consistently been challenging for non-face-to-face education content, as noted previously [9,33]. Considering the nature of pediatric physical education content, it is common for supervisors to be parents. Therefore, incorporating explanations specifically directed at parents would further enhance the educational value of the content.

Low scores for the patient selection and eligibility-related items for i-CONTENT and CONTENT were attributed to the lack of information on target age groups. Consequently, the lack of age-related information led to lower scores for the “Competences and setting” item for CONTENT. On the other hand, high scores were obtained for the “Dosage parameters (frequency, intensity, time)”, “Type of exercise”, and “Safety of the exercise program” items of i-CONTENT and the “Rationale” item of CONTENT. However, the “Content” item of CONTENT received a low satisfaction rating. This suggests that while the general information about exercise such as purpose and method, type, and potential complications were clearly mentioned, there was a lack of individualized exercise options, which showed similar results to the previous study about home exercises [34]. To address these limitations, recent studies have explored digital adherence technologies [35,36], wearable sensors designed for physical performance monitoring [37], app-based interfaces [38,39], and hybrid models [40] to improve adherence and personalize exercise programs. In particular, based on prior research on the positive effects of social media exercise content, it offers unique advantages that conventional exercise education programs cannot replicate, such as strong initial engagement through experience sharing or the influencer effect [41], easy accessibility, a wide variety of exercise options [42], and a sense of accountability through social feedback [43]. It is expected that a detailed strategy incorporating these strengths could address the limitations identified in this study.

The correlation analysis of the total scores of the quantitative evaluation tools for video content and other qualitative evaluation tools showed no statistically significant correlations. This finding suggests no correlation between video popularity and the qualitative aspects of video content or exercise education programs, which is consistent with previous quality analyses of YouTube-based content [32,44]. In the correlation analysis of the total scores of video quality and the exercise education program tools, a moderate correlation was observed, which could be explained by the extensive research on the impact of multimedia components on educational outcomes [45]. However, no significant correlation was observed for the scores of GQS, which reflects overall satisfaction with flow and quality; however, the significant difference in the satisfaction ratios between the two sections of this study could have influenced the results.

The correlation analysis between two qualitative assessment tools revealed that video information about uncertainty and reliability (m-DISCERN) affects exercise program safety (i-CONTENT). Additionally, the aims of the videos (m-DISCERN) were strongly correlated with the type (i-CONTENT) and rationale (CONTENT) of the exercise program. Furthermore, purpose-specific detailed exercise education content (CONTENT) was strongly related to information balance (m-DISCERN) and moderately related to video flow (GQS). However, the satisfaction rates of all items mentioned in this paragraph were below 20%, and the remaining items showed no significant correlations among them, indicating that video quality tools may not effectively assess exercise program quality, particularly in relation to eligibility, dosage, and adherence.

### 4.2. Limitations

This study has several strengths and limitations. One strength is its focus on the qualitative analysis of YouTube-based educational exercise programs, providing valuable insights into the content and structure of digital educational tools for children. However, a limitation of this study is that video popularity may have been influenced by the potential manipulation of view counts through certain applications, and the study design lacked an analysis of their clinical effects. Furthermore, the content analyzed was limited to the YouTube platform. Future research could develop an optimal tool for evaluating digital content across a broader range of platforms, including TikTok, Instagram, and Facebook, and further investigate the clinical impact of these contents on physical activity in children and adolescents.

## 5. Conclusions

YouTube-based exercise education content targeting children has educational potential but faces limitations in terms of outcome assessment, presence of a qualified supervisor, target eligibility, adherence, and dosage parameters. Considering these aspects, YouTube-based content currently falls short as a substitute for in-person education. To supplement this, future integration with digital adherence technologies, hybrid interventions, and greater involvement of professional healthcare providers is necessary.

Moreover, the quality evaluation tools for video content and exercise programs were unable to evaluate YouTube-based content sufficiently. Considering that YouTube-based content is an inevitable trend that will continue to expand, it is necessary to develop a dedicated tool that can evaluate YouTube-based exercise programs for children.

## Figures and Tables

**Table 1:** Basic characteristics and quantitative analysis of video contents.

Variables	Value
Total videos (n)	126
Health professional uploader	13 (10.31)
Exercise type	
Aerobic	4 (3.17)
Bone and muscle strengthening	9 (7.14)
Multicomponent physical activity	106 (84.13)
Other	7 (5.56)
Video duration	813.50 ± 411.37
Likes	315.50 (58.25–4276.00)
Dislikes	9.00 (0.00–405.8)
Views	54,640 (7324–599,011)
VPI	252.00 (31.25–826.25)

Values are shown as number (percentage), mean ± standard deviation, or median (IQR1–IQR3). Abbreviations: IQR, interquartile range; VPI, Video Power Index.

**Table 2:** Quality analysis of video contents.

Evaluation Tool	Value
m-DISCERN total score	3.28 ± 1.01
Unreliable	23 (18.25)
Reliable	103 (81.75)
GQS total score	3.29 ± 1.01
High	30 (23.80)
Moderate	53 (42.06)
Low	43 (34.12)

Values are shown as number (percentage) or mean ± standard deviation. Abbreviations: m-DISCERN, modified DISCERN; GQS, global quality scale.

**Table 3:** Results of quality analysis of educational programs.

Evaluation Tool	Value
i-CONTENT total score	2.62 ± 0.77
Low risk of ineffectiveness	0 (0)
Moderate risk of ineffectiveness	8 (6.35)
High risk of ineffectiveness	118 (93.65)
CONTENT total score	1.53 ± 1.25
Effective	2 (1.6)
Ineffective	124 (98.41)
CERT total score	4.50 ± 2.36

Values are shown as number (percentage) or mean ± standard deviation. Abbreviations: i-CONTENT, International CONsensus on Therapeutic Exercise aNd Training; CONTENT, CONsensus on Therapeutic Exercise aNd Training; CERT, Consensus on Exercise Reporting Template.

**Table 4:** Correlation results of video popularity and qualitative assessments for video content and exercise education.

Variable/Tool		Quantitative Assessment				
		Like	Dislike	View Ratio	View	VPI
Video content quality	m-DISCERN	0.05	0.07	0.08	0.07	0.07
	GQS	−0.06	−0.12	0.07	−0.03	0.05
Exercise program quality	i-CONTENT	−0.01	−0.1	0.05	−0.01	0.09
	CONTENT	0.03	0.05	0.02	0.04	0.04
	CERT	0.07	0.07	0.06	0.09	0.06

A Pearson correlation analysis was used to derive these data. Abbreviation: m-DISCERN, modified DISCERN; GQS, global quality scale; i-CONTENT, International CONsensus on Therapeutic Exercise aNd Training; CONTENT, CONsensus on Therapeutic Exercise aNd Training; CERT, Consensus on Exercise Reporting Template; VPI, Video Power Index.

## Data Availability

The data sets generated in the present study are available by request from the corresponding author.

## Supplementary Materials

The following supporting information can be downloaded at: https://www.mdpi.com/article/10.3390/healthcare13050560/s1. Table S1: Keywords applied in YouTube search of this study. Table S2: Information about types of exercise for included videos in this study.
